# Prior Thrombectomy Does Not Affect the Surgical Complication Rate of Decompressive Hemicraniectomy in Patients with Malignant Ischemic Stroke

**DOI:** 10.1007/s12028-023-01820-3

**Published:** 2023-08-28

**Authors:** Johannes Walter, O. T. Alhalabi, S. Schönenberger, P. Ringleb, D. F. Vollherbst, M. Möhlenbruch, A. Unterberg, J.-O. Neumann

**Affiliations:** 1 Department of Neurosurgery, Heidelberg University Hospital, Im Neuenheimer Feld 400, 69120 Heidelberg, Germany; 2 Department of Neurology, Heidelberg University Hospital, Heidelberg, Germany; 3 Department of Neuroradiology, Heidelberg University Hospital, Heidelberg, Germany

**Keywords:** Decompressive hemicraniectomy, Decompressive craniectomy, Stroke, Malignant stroke, Thrombectomy, Mechanical revascularization, Mechanical recanalization

## Abstract

**Background:**

Even though mechanical recanalization techniques have dramatically improved acute stroke care since the pivotal trials of decompressive hemicraniectomy for malignant courses of ischemic stroke, decompressive hemicraniectomy remains a mainstay of malignant stroke treatment. However, it is still unclear whether prior thrombectomy, which in most cases is associated with application of antiplatelets and/or anticoagulants, affects the surgical complication rate of decompressive hemicraniectomy and whether conclusions derived from prior trials of decompressive hemicraniectomy are still valid in times of modern stroke care.

**Methods:**

A total of 103 consecutive patients who received a decompressive hemicraniectomy for malignant middle cerebral artery infarction were evaluated in this retrospective cohort study. Surgical and functional outcomes of patients who had received mechanical recanalization before surgery (thrombectomy group, *n* = 49) and of patients who had not received mechanical recanalization (medical group, *n* = 54) were compared.

**Results:**

The baseline characteristics of the two groups did significantly differ regarding preoperative systemic thrombolysis (63.3% in the thrombectomy group vs. 18.5% in the medical group, *p* < 0.001), the rate of hemorrhagic transformation (44.9% vs. 24.1%, *p* = 0.04) and the preoperative Glasgow Coma Score (median of 7 in the thrombectomy group vs. 12 in the medical group, *p* = 0.04) were similar to those of prior randomized controlled trials of decompressive hemicraniectomy. There was no significant difference in the rates of surgical complications (10.2% in the thrombectomy group vs. 11.1% in the medical group), revision surgery within the first 30 days after surgery (4.1% vs. 5.6%, respectively), and functional outcome (median modified Rankin Score of 4 at 5 and 14 months in both groups) between the two groups.

**Conclusions:**

A prior mechanical recanalization with possibly associated systemic thrombolysis does not affect the early surgical complication rate and the functional outcome after decompressive hemicraniectomy for malignant ischemic stroke. Patient characteristics have not changed significantly since the introduction of mechanical recanalization; therefore, the results from former large randomized controlled trials are still valid in the modern era of stroke care.

**Supplementary Information:**

The online version contains supplementary material available at 10.1007/s12028-023-01820-3.

## Introduction

The acute treatment of ischemic stroke due to large vessel occlusion has been revolutionized by the introduction of mechanical recanalization. In the years 2015 and 2016, six large prospective multicenter randomized controlled trials independently showed that the use of mechanical recanalization techniques significantly improved reperfusion rates and functional outcomes 3 months after acute stroke without increasing the rates of serious adverse events, e.g., intracranial hemorrhage [1–6]. As a consequence, indications of mechanical recanalization procedures for large vessel occlusion have been expanded, and rates of thrombectomies have significantly increased [7, 8]. Although the Randomized Asessment of Rapid Endovascular Treatment of Ischemic Stroke (ESCAPE) and Thrombectomy within 8 Hours after Symptom Onset in Ischemic Stroke (REVASCAT) trials both report relatively low rates of decompressive hemicraniectomies following mechanical recanalization procedures (e.g., 2 in 315 patients in ESCAPE, 9 in 206 patients in REVASCAT), there are conflicting data on whether the advances in acute stroke treatment have subsequently led to a decreased necessity for decompressive hemicraniectomies due to malignant edema formation in general [1, 6–11]. Therefore, decompressive hemicraniectomy remains a valuable rescue measure for malignant courses of ischemic stroke, as in the past, it has been shown that surgical decompression can significantly reduce morbidity and mortality in these cases [12, 13]. However, it remains unclear whether the characteristics of the patients requiring decompressive hemicraniectomy after ischemic stroke have changed following the addition of thrombectomy to the treatment armamentarium and, therefore, whether the conclusions drawn from the large multicenter randomized controlled trials evaluating the effects of decompressive hemicraniectomy in the context of malignant courses of ischemic stroke remain valid in times of modern stroke care.

Furthermore, mechanical recanalization procedures are typically accompanied by prior systemic thrombolysis therapy and/or periprocedural administration of antiplatelets or anticoagulants [1–3, 6]. The effect of a prior thrombolytic bridging therapy on hemorrhagic complications of stroke or mechanical recanalization procedures has been studied extensively, and meta-analyses have largely shown that prior bridging thrombolysis does not seem to increase hemorrhagic complications [14–18]. However, hemorrhagic events, such as epidural or intraparenchymal bleedings due to decompression, are among the most frequent surgical complications of decompressive hemicraniectomy. In addition, recent studies have shown that the use of antiplatelets and anticoagulants significantly increases the rate of hemorrhagic complications of decompressive hemicraniectomy for the treatment of intracerebral hemorrhage [19–21]. Yet the effect of a mechanical recanalization procedure with or without prior bridging thrombolysis on the surgical complication rate of a subsequent decompressive hemicraniectomy for malignant cerebral edema remains understudied.

Therefore, we evaluated the patient characteristics and studied the correlation of bridging thrombolysis as well as mechanical recanalization with the rate of early surgical complications and functional outcome after decompressive hemicraniectomy for malignant ischemic stroke due to large vessel occlusion in the anterior circulation.

## Methods

### Data Collection

The data analyzed in this study were collected retrospectively. Information on baseline characteristics and treatment data was collected from patient charts, intensive care unit (ICU) charts, radiology reports, and operation/procedure reports. Outcome data were collected from outpatient clinic reports as well as from the local stroke registry. The dimension of the decompression was manually assessed based on the bone window of the first postoperative computed tomography (CT) scan by two independent raters (JW and OTA) experienced in assessing CT scans of the cranium. Prior to data collection, the study protocol was approved by the local standing committee on ethical practice of the University of Heidelberg (approval number S-667/2021).

### Patients

A total of 103 consecutive patients with ischemic stroke due to an occlusion of the intracranial internal carotid artery, middle cerebral artery, anterior cerebral artery, or a combination thereof who received a decompressive hemicraniectomy at our academic medical center between January 1, 2015, and December 31, 2021, were analyzed. Patients who received a decompressive craniectomy of the posterior fossa due to ischemic stroke of the posterior circulation were excluded from the analysis. The initial diagnosis was established based on clinical assessment and imaging findings provided by native CT, CT angiography, and CT perfusion scans, followed by digital subtraction angiography. The decision to perform thrombectomy was made by the treating senior neuroradiologist based on the current guidelines and on a case-by-case basis for all patients included in the study. In general, the major indication for performing thrombectomy was large vessel occlusion (distal internal carotid artery, carotid T, M1 or proximal M2 segment of the middle cerebral artery). Additional factors influencing the decision were clinical and radiological stroke severity, patient age, premorbid functional status, and the patient’s living will. The mechanical recanalization procedures were conducted by the treating senior neuroradiologist using standard treatment techniques (stent retriever thrombectomy or direct aspiration) under general anesthesia or conscious sedation. The standard interventional approach includes a femoral access with an 8F access sheath using a triaxial catheter system comprising an 8F guiding catheter, a 5F or 6F intermediate/aspiration catheter, and a microcatheter microwire system for stent retriever thrombectomy under continuous aspiration or direct aspiration. The indication for performing decompressive hemicraniectomy was based on an interdisciplinary discussion of a senior neurosurgeon and the treating senior neurologist on an individual basis. Surgery was performed using a reversed question mark incision by removing a fronto-temporo-parietal bone flap of at least 12 cm in diameter, with a subsequent opening of the dura, and performing nonrestricting dura expansion followed by placement of two subgaleal drains, which were removed 24–48 h postoperatively.

### Clinical Assessment

Patients were clinically assessed using the Glasgow Coma Scale (GCS) as well as the National Institutes of Health Stroke Scale (NIHSS). The GCS is used to evaluate a patient’s level of consciousness after a brain injury. The total score is the sum of three subscores evaluating eye opening, speech, and motor function; it ranges from 3 to 15, with higher scores indicating less severe impairment of the level of consciousness [22]. The NIHSS is a tool used to objectively quantify clinical impairment caused by a stroke. Its total score is calculated by adding the subscores of 11 different items, such as speech, level of consciousness, and motor function of the legs and arms; it ranges from 0 to 42 points, with higher scores indicating more severe clinical impairment [23].

### Outcome Assessment

Functional outcome was assessed using the modified Rankin Scale (mRS), which rates a patient’s functional status on a scale ranging from 0 to 6 points, with higher scores indicating more severe functional impairment. An mRS score of 0 indicates that the patient does not experience any symptoms at all, whereas bedridden patients receive an mRS score of 5. An mRS score of 6 means that the patient has died [24]. Premorbid functional status was obtained from relatives whenever possible. The mRS was scored by either the treating physician or a trained study nurse at the time of cranioplasty (first follow-up) and as part of the 1-year follow-up visit at the outpatient clinic.

### Statistical Analysis

All continuous variables are summarized as mean ± SEM for normally distributed data or as median and range of data points for nonparametric data. Continuous variables were compared using *t*-tests for normally distributed variables or the Mann–Whitney *U*-test for nonnormally distributed variables. Discrete variables were compared using the χ^2^ test or Fisher’s exact test, when appropriate. To examine possible effects of covariates other than thrombectomy or medical treatment on the surgical revision rate, a binomial logistic regression analysis was performed. Thrombectomy (mechanical recanalization), systemic thrombolysis, pathological coagulation parameters, hemorrhagic transformation, time from symptom onset to surgery, and the presence of cardiovascular disease in the past medical history of patients were included as independent variables, and the surgical revision rate was the dependent variable. Statistical significance was predefined as *p* ≤ 0.05. All statistical analyses were conducted using a data analysis software package (Prism 8, GraphStat Technologies). The binomial regression analysis was performed using R.

## Results

On average, patients were in their mid-fifties (54.0 and 54.8 years in the thrombectomy and medical treatment groups, respectively) and predominantly male (38.8% and 42.6% female patients, respectively). Patients in the thrombectomy and medical treatment groups had an average of 1.7 and 2.0 cardiovascular diseases (arterial hypertension, coronary heart disease, congestive heart failure, cardiac arrythmia, peripheral artery disease, prior stroke, prior myocardial infarction), respectively. About one third of the patients were on antiplatelets prior to surgery (28.6% and 33.3% in the thrombectomy and medical treatment groups, respectively), whereas only 4.1% and 9.3% of the patients, respectively, were on anticoagulants. Preoperatively, no patient had a prolonged activated prothrombin time, and in only 10.2% and 7.4% of the cases, respectively, the international normalized ratio was more than 1.2. Although the initial NIHSS score (the first documented NIHSS score at either a referring hospital or our medical center) was lower in the thrombectomy group (14 vs. 17, *p* = 0.08), there were no differences in the NIHSS and GCS scores at admission to our hospital between the two groups (median NIHSS scores of 20 and 21, median GCS scores of 11 and 12, respectively). In about one third of the patients in the thrombectomy group, more than one vascular territory was affected (34.7%), and in about half of the patients in this group (44.9%), hemorrhagic transformation of the infarction (based on radiological findings and defined as any hemorrhagic infarction or parenchymal hematoma) could be detected preoperatively, whereas in the medical treatment group, more than one vascular territory was affected in only 18.5% of the patients, and the infarction showed hemorrhagic transformation in only 24.1% of the patients, which accounted for a statistically significant difference (*p* = 0.04). Interestingly, left-sided decompression was performed in only about one third of the patients in both groups (34.7% and 29.6%, respectively). Systemic thrombolysis was performed in two thirds of the patients who later received mechanical recanalization, whereas in the medical treatment group, only 18.5% of the patients received systemic thrombolysis prior to surgery, which accounted for a statistically significant difference between the two groups (*p* < 0.001). Preoperatively, NIHSS and mRS scores did not significantly differ between the two groups (median NIHSS scores of 25 and 21, respectively; median mRS score of 5 in both groups), whereas patients with prior thrombectomy had significantly lower median GCS scores (7 vs. 12, *p* = 0.04). Preoperative pupillary abnormalities were present in twice as many patients (24.5% vs. 11.1%), and time from the onset of symptoms to surgical decompression was shorter (31.4 vs. 42.3 h) in the thrombectomy group compared with the medical treatment group; however, these differences were not statistically significant. Table 1 summarizes the baseline characteristics.

**Table 1:** Baseline characteristics

	Total (*n* = 103)	Thrombectomy (*n* = 49)	Medical treatment (*n* = 54)	*p* value^a^
Age (y), mean ± SD	54.4 ± 1.2	54.0 ± 1.9	54.8 ± 1.5	0.73
Female sex	40.8% (42/103)	38.8% (19/49)	42.6% (23/54)	0.85
Cardiovascular diseases, mean ± SD	1.86 ± 0.2	1.7 ± 0.2	2.0 ± 0.3	0.80
Antiplatelet use	31.1% (32/103)	28.6% (14/49)	33.3% (18/54)	0.76
Anticoagulant use	6.8% (7/103)	4.1% (2/49)	9.3% (5/54)	0.52
aPTT > 35 s	0% (0/103)	0% (0/49)	0% (0/54)	n.a
INR > 1.2	8.7% (9/103)	10.2% (5/49)	7.4% (4/54)	0.88
pmRS, median (range)	0 (0–2)	0 (0–2)	0 (0–2)	0.48
Initial NIHSS score, median (range)	15 (3–38)	14 (3–33)	17 (5–38)	0.08
NIHSS score at admission, median (range)	20 (8–38)	20 (8–38)	21 (10–38)	0.32
GCS score at admission, median (range)	12 (3–15)	11 (3–15)	12 (3–15)	0.95
> 1 vascular territory affected	26.2% (27/103)	34.7% (17/49)	18.5% (10/54)	0.10
Occlusion of ICA	9.4% (48/103)	46.9% (23/49)	46.3% (25/54)	1.0
Hemorrhagic transformation^b^	34.0% (35/103)	44.9% (22/49)	24.1% (13/54)	**0.04**
Left-sided decompression	32.0% (33/103)	34.7% (17/49)	29.6% (16/54)	0.74
Systemic thrombolysis	39.8% (41/103)	63.3% (31/49)	18.5% (10/54)	** < 0.001**
Preoperative mRS score, median (range)	5 (3–5)	5 (3–5)	5 (4–5)	0.18
Preoperative NIHSS score, median (range)	21 (8–38)	25 (13–38)	21 (8–38)	0.09
Preoperative GCS score, median (range)	10 (3–15)	7 (3–15)	12 (3–15)	**0.04**
Pupillary abnormality present	17.5% (18/103)	24.5% (12/49)	11.1% (6/54)	0.13
Time to decompression (h), mean ± SD	36.2 ± 3.2	31.4 ± 3.4	42.3 ± 5.7	0.06

Bold values indicate statistically significant *p*-values ≤ 0.05

aPTT, activated prothrombin time, GCS, Glasgow Coma Scale, ICA, internal carotid artery, INR, international normalized ratio, mRS, modified Rankin Scale, pmRS, premorbid modified Rankin Scale score (mRS score before acute stroke), n.a., not applicable, NIHSS, National Institutes of Health Stroke Scale

^a^Thrombectomy vs. medical treatment

^b^Preoperative hemorrhagic transformation of the stroke

### Surgical Complications and ICU Stay

The average decompression size was 12.9 cm in both groups. Overall, the rates of surgical complications and revision surgeries were low (10.7% and 4.9%, respectively), and there was no difference in the rates of surgical complications (10.2% vs. 11.1%) and revision surgeries (4.1% vs. 5.6%) between the two groups. Also, there was no difference between the groups regarding secondary progression of hemorrhagic transformation of the stroke after decompressive hemicraniectomy (44.4% vs. 39%, respectively). The length of the ICU stay was similar in both groups (11.4 vs. 12.1 days), and most patients were ventilated at discharge (75.7% and 63.8%, respectively). The median NIHSS score was significantly higher (35 vs. 25, *p* = 0.03) and the median GCS score was significantly lower (4 vs. 10, *p* = 0.15) at discharge from the ICU in the thrombectomy group, whereas the median mRS score at discharge and in-hospital mortality did not significantly differ between the two groups (median mRS score of 5 in both groups, in-hospital mortality of 24.5% and 13.0%, respectively). Tables 2 and 3 summarize the data on early outcome, surgical complications, and indications for surgical revision.

**Table 2:** Surgical complications and ICU stay

	Total (*n* = 103)	Thrombectomy (*n* = 49)	Medical treatment (*n* = 54)	*p* value^a^
Maximal diameter of decompression (cm), mean ± SD	12.9 ± 0.1	12.9 ± 0.1	12.9 ± 0.2	0.90
Surgical complication	10.7% (11/103)	10.2% (5/49)	11.1% (6/54)	0.87
Surgical revision	4.9% (5/103)	4.1% (2/49)	5.6% (3/54)	0.91
Hemorrhagic transformation^b^	61.2% (63/103)	69.4% (34/49)	53.7% (29/54)	0.15
Hemorrhagic progression^c^	41.2% (28/68)	44.4% (12/27)	39.0% (16/41)	0.85
ICU stay (d), mean ± SD	11.8 ± 0.5	11.4 ± 0.7	12.1 ± 0.7	0.46
mRS score at discharge, median (range)	5 (3–6)	5 (3–6)	5 (4–6)	0.13
NIHSS score at discharge, median (range)	30 (6–38)	35 (6–38)	25 (8–38)	**0.03**
GCS score at discharge, median (range)	6 (3–15)	4 (3–15)	10 (3–15)	0.15
Intubated at discharge	69.0% (58/84)	75.7% (28/37)	63.8% (30/47)	0.35
In-hospital mortality	18.4% (19/103)	24.5% (12/49)	13.0% (7/54)	0.21

Bold value indicates statistically significant *p*-value ≤ 0.05

﻿GCS, Glasgow Coma Scale, ICU, intensive care unit, mRS, modified Rankin Scale, NIHSS, National Institutes of Health Stroke Scale

^a^Thrombectomy vs. medical treatment

^b^Hemorrhagic transformation of the stroke after decompressive hemicraniectomy

^c^Progression from no preoperative hemorrhagic transformation to postoperative hemorrhagic transformation of the stroke

**Table 3:** Surgical complications and indications for surgical revision

Thrombectomy (*n* = 5)	Medical treatment (*n* = 6)
Epidural hematoma (*n* = 3)	Epidural hematoma (*n* = 3, surgical revision in all cases)
Subdural hematoma (*n* = 1, revised surgically)	Subdural hematoma (*n* = 3)
Space-occupying intracerebral hemorrhage (*n* = 1, revised surgically)	

As expected, binomial regression analysis revealed that mechanical recanalization prior to surgical decompression did not correlate with surgical complications. Interestingly, although overrepresented in the thrombectomy group, systemic thrombolysis was associated with a lower surgical complication rate. Indeed, of 41 patients undergoing systemic thrombolysis, only one patient experienced a surgical complication, compared to 10 of 62 patients who did not receive systemic thrombolysis. Table 4 and Supplemental Table 1 summarize the results of the binomial regression analysis with surgical complication (Table 4) and surgical revision (Supplemental Table 1) as dependent variables.

**Table 4:** Output summary of binomial regression analyses with surgical complication as the dependent variable and the listed covariates as independent variables

	Estimated SD^a^	SE	*z*-value^b^	Adjusted *p*^c^
Thrombolysis	− 3.487	1.580	− 2.2070	**0.027**
Mechanical recanalization	1.931	1.255	1.5386	0.124
Hemorrhagic transformation	1.052	1.119	0.9401	0.347
Antiplatelet use	0.882	1.201	0.7344	0.463
Anticoagulant use	− 19.284	21.767.584	− 0.0009	0.999
INR > 1.2	− 18.748	16.596.773	− 0.0011	0.999
CVD > 2	0.366	1.150	0.3183	0.750
Time from onset to surgery	− 0.027	0.023	− 1.1739	0.241
Preoperative GCS score < 9	− 1.708	1.254	− 1.3620	0.173

Bold value indicates statistically significant *p*-value ≤ 0.05

CVD, cardiovascular disease, GCS, Glasgow Coma Scale, INR, international normalized ratio

^a^Positive values indicate negative correlation; negative values indicate positive correlation

^b^Ratio of estimated SD and SE

^c^*p* value of Wald test adjusted for multiple testing

### Functional Outcome

The average time to the first (4.8 and 4.4 months, respectively) and second follow-up (14.6 and 15.1 months, respectively) did not differ between the two groups. Whereas only 4.8% of the patients missed the first follow-up, data on functional outcome were only available for 39.3% of the patients at the second follow-up. The median mRS score was 4 in both groups at both time points and, therefore, did not differ between the groups, which was also true for the proportion of patients with a favorable functional outcome, which was defined as an mRS score of 3 points or less (17.1% and 15.6% and 41.2% and 43.8% of the patients in the thrombectomy and medical treatment groups at the first and second follow-up, respectively). Table 5 summarizes the data on functional outcome.

**Table 5:** Functional outcome at first and second FU

	Total (*n* = 103)	Thrombectomy (*n* = 49)	Medical treatment (*n* = 54)	*p* value^a^
Time to first FU (months), mean ± SD	4.6 ± 0.3	4.8 ± 0.4	4.4 ± 0.4	0.13
mRS score at first FU, median (range)	4 (2–5)	4 (2–5)	4 (3–5)	0.12
mRS score < 4 at first FU	16.3% (13/80)	17.1% (6/35)	15.6% (7/45)	0.91
Loss-to-FU at first FU	4.8% (4/84)	5.4% (2/37)	4.3% (2/47)	1.0
Time to second FU (months), mean ± SD	13.6 ± 1.5	14.6 ± 1.5	15.1 ± 2.3	0.85
mRS score at second FU, median (range)	4 (1–6)	4 (1–5)	4 (2–6)	0.85
mRS score < 4 at first FU	42.4% (14/33)	41.2% (7/17)	43.8% (7/16)	0.84
Loss-to-FU at second FU	60.7% (51/84)	54.1% (20/37)	66.0% (31/47)	0.38

FU, follow-up, mRS, modified Rankin Scale

^a^Thrombectomy vs. medical treatment

## Discussion

### Prior Systemic Thrombolysis and Mechanical Recanalization Do Not Affect the Surgical Complication Rate

In our study, the rates of surgical complications, revision surgeries, and secondary hemorrhagic transformation of the stroke due to surgical decompression did not differ between patients who had received a mechanical recanalization procedure prior to decompressive hemicraniectomy and patients treated conservatively, even though a significantly higher proportion of patients had received a systemic antifibrinolytic treatment prior to surgical intervention in the thrombectomy group (Table 2). Furthermore, even though the analysis needs to be interpreted cautiously because of the relatively small number of observed outcomes, a mechanical recanalization procedure prior to surgical decompression did not correlate with surgical complications and the need for revision surgery in a binomial regression analysis (Table 4 and Supplemental Table 1). Interestingly, systemic thrombolysis was associated with a lower complication rate. Given the retrospective nature of this analysis and the sample size, it would be conceivable to at least argue that neither systemic antifibrinolytic treatment nor interventional thrombectomy negatively affect the rate of surgical complications of decompressive hemicraniectomy for malignant courses of ischemic stroke. This might be explained by the fact that the biologic half-life of recombinant tissue plasminogen activator (rTPA), the standard agent used for systemic antifibrinolytic therapy, is only 5 min, whereas the average time from the onset of symptoms to surgical decompression was 36 h in our study [25] (Table 1). Therefore, even though it has been reported that the thrombolytic effect of rTPA might last up to 48 h, one would not expect a higher risk of hemorrhagic complications of decompressive hemicraniectomy more than 30 h after systemic administration of rTPA, which is backed up by our data indicating a very low rate of impaired coagulation directly prior to surgical decompression (no patients with impaired activated prothrombin time in both groups, 10.2% and 7.4% of the patients with impaired international normalized ratio in the thrombectomy and medical treatment group, respectively; Table 1). This also indicates that periinterventional medication administered in the context of mechanical revascularization does not significantly affect intraoperative coagulation and, therefore, the risk of intraoperative bleeding or postoperative hemorrhagic complications of a subsequent decompressive hemicraniectomy.

Despite the fact that prior systemic thrombolysis was significantly associated with lower surgical complication rates, we did observe a significantly increased rate of hemorrhagic transformation of the stroke prior to surgery in the thrombectomy group, possibly diminishing the observed positive effect of thrombolysis. This corresponds to the results of recent studies showing that intravenous antifibrinolytic treatment prior to mechanical revascularization might increase the risk of hemorrhagic transformation of the stroke [17, 26].

Our observations concerning the risk profile of decompressive hemicraniectomy for malignant ischemic stroke after mechanical recanalization are well in line with those of other studies that also could not detect a correlation between a prior revascularization treatment and the rate of surgical complications [27].

### The Outcome After Decompressive Hemicraniectomy for Malignant Stroke Is Not Affected by Prior Mechanical Recanalization

We did not observe any difference in mortality or functional outcome between the population of patients who required decompressive hemicraniectomy despite prior thrombectomy and medically treated patients (Tables 2 and 5). This is remarkable considering the fact that patients in the thrombectomy group were clinically more impaired prior to surgery compared with the medically treated patients (i.e., median preoperative NIHSS score of 25 vs. 21 and 24.5% vs. 11.1% of the patients with pupillary abnormalities, respectively; Table 1). This might be explained by multiple factors:

1. Neither prior systemic thrombolysis nor a prior recanalization procedure increased the surgical complication rate, which might have affected the functional outcome.
2. The recanalization procedure did not lead to any delay of decompressive hemicraniectomy (in fact, patients in the thrombectomy group received surgical decompression even earlier than medically treated patients in our study), and therefore decompressive hemicraniectomy was still performed well within the recommended time window of 36 to 48 h after the onset of symptoms [12, 13].
3. The need for decompressive hemicraniectomy for intractable increased intracranial pressure despite successful revascularization might be due to severe early edema rather than progression of ischemic stroke, possibly resulting in more salvageable tissue and, therefore, a higher likelihood of improving functional outcome with performing a decompressive hemicraniectomy.
4. Prior successful mechanical revascularization might support the beneficial effect of decompressive hemicraniectomy on perfusion of the ischemic penumbra. Of note, in 30.6% of the analyzed cases, restoration of anterograde flow in the target vessels could not be achieved by mechanical thrombectomy, and because the data analysis was performed as an intention-to-treat analysis, patients with failed recanalization procedures did not cross over to the medical treatment arm. Therefore, potential positive synergistic effects of mechanical revascularization and decompressive hemicraniectomy might even be underestimated by our study.

### Patient Characteristics Have Not Changed Significantly Since the Introduction of Thrombectomy

The baseline patient characteristics of the thrombectomy group in our study were similar to those of the patients in the pivotal randomized controlled trials of decompressive hemicraniectomy as a treatment for malignant courses of ischemic stroke regarding sex (predominantly male patients), age (mean age of around 55 years), clinical (mean preoperative NIHSS score of 25) as well as radiological (more than one vascular territory affected in about one third of the patients) features, and time from the onset of symptoms to decompression (around 30 h) [28]. Also, early (after 3–6 months) and long-term functional outcomes (after 12 months) were similar; therefore, the conclusions drawn from the large randomized controlled trials on decompressive hemicraniectomy in malignant stroke, which were conducted before mechanical recanalization became part of the routine stroke treatment, still seem relevant and valid in times of modern stroke care [12, 13]. This observation is also well in line with other studies evaluating the role of decompressive hemicraniectomy in times of mechanical recanalization that share similar patient characteristics, functional outcome data, and conclusions with our study. Göttsche et al. [29] found a decrease the rate of decompressive hemicraniectomies between 2009 and 2017, but patient characteristics remained unchanged and similar to those in our study regarding age (54 years in both studies), sex (39% vs. 41% female), initial NIHSS score (18 vs. 20), and time to decompression (around 30 h in both studies). However, only 32 patients had received mechanical thrombectomy prior to decompressive hemicraniectomy in this study, and rates of surgical complications and revision rates were not reported. In contrast to the study by Göttsche et al., Alzayiani et al. [27] focused on the effect of prior mechanical recanalization or systemic thrombolysis on surgical complications of decompressive hemicraniectomy. The study population was comparable to our cohort regarding demographics (age of 51 vs. 54 years, 44% vs. 41% female patients) and preoperative neurological status (e.g., GCS score of 10 in both studies). Although the rate of revision surgery was significantly higher compared with that in our study, no association between either prior systemic thrombolysis or mechanical recanalization and surgical complications could be detected. However, the conclusions need to be interpreted cautiously because more than 100 of the initially screened 152 patients were excluded prior to data analysis.

### Limitations

Several limitations need to be considered when interpreting the presented data. First of all, the study was conducted in a retrospective manner; therefore, there was no randomization of study participants to either treatment group, and baseline parameters were not exactly balanced, which limits generalizability of the study results. Furthermore, even though we report on the largest cohort of patients with prior mechanical recanalization before decompressive hemicraniectomy for ischemic stroke due to large vessel occlusion, patient numbers are still limited because of the monocentric nature of the study. This is especially true for the data on functional outcome at the second follow-up (as data on functional status were only available for 40% of the patients initially enrolled in the study) and the relatively small number of observed outcomes (surgical complications and revision surgery) in the binomial regression analysis. Therefore, cautious interpretation of these data is indicated, and pooling of retrospective data from different centers, as well as prospective multicenter approaches, such as the initiation of a prospective multicenter registry, is desirable to provide more detailed insight into the topic in the future.

## Conclusions

Systemic antifibrinolytic treatment and mechanical revascularization procedures prior to decompressive hemicraniectomy for malignant courses of ischemic stroke due to large vessel occlusion of the anterior circulation do not affect surgical complication rates or functional outcome. Conclusions drawn from the pivotal trials of decompressive hemicraniectomy as treatment for malignant ischemic stroke are still valid in times of modern acute stroke, as the introduction of mechanical recanalization techniques has not significantly altered the characteristics of patients requiring decompressive hemicraniectomy for intractably increased intracranial pressure. In the future, multicenter approaches should be pursued to improve data quality and gain more detailed insight into the topic.

### Supplementary Information

Below is the link to the electronic supplementary material.

Supplementary file 1 (DOCX 14 KB)

## Data Availability

The data sets used and/or analyzed in the current study are available from the corresponding author upon reasonable request.
