# Supplementary material for: Prior Thrombectomy Does Not Affect the Surgical Complication Rate of Decompressive Hemicraniectomy in Patients with Malignant Ischemic Stroke
Source: Neurocrit Care. 2023 Aug 28;40(2):698–706. doi: 10.1007/s12028-023-01820-3 (PMC10959817; doi:10.1007/s12028-023-01820-3)
Supplement: Supplementary file 1 — (DOCX 14 KB) [file 12028_2023_1820_MOESM1_ESM.docx]

**Binomial Regression Analysis with surgical revision**

**as the dependent variable**

|  | **Estimate Std.** | | **Std. Error** | **z-value** | **Adj-p** |
| --- | --- | --- | --- | --- | --- |
| **Thrombolysis** | -2.67434 | | 1.18432 | -2.258 | **0.024** |
| **Mechanical recanalization** | 0.70438 | 0.91938 | | 0.766 | 0.444 |
| **Hemorrhagic transformation** | 0.29287 | 1.00596 | | 0.291 | 0.771 |
| **Antiplatelet Use** | 0.22795 | 1.02534 | | 0.222 | 0.824 |
| **Anticoagulant Use** | 1.0326 | 1.64477 | | 0.628 | 0.530 |
| **INR>1.2** | -16.68019 | 2380.83363 | | -0.007 | 0.994 |
| **CVD> 2** | -0.19711 | 0.99209 | | -0.199 | 0.843 |
| **Time from onset to surgery** | -0.01969 | 0.02029 | | -0.971 | 0.332 |

**Table 4:** *Output summary of binomial regression analyses with surgical revision as the dependent variable and the listed co-variates as independent variables.* INR: international normalized ratio. CVD; cardiovascular disease. GCS: Glasgow Coma Scale. Estimate Std.: estimated standard deviation (positive values indicate negative correlation; negative values indicate positive correlation). Std. Error: standard error. z-value: ratio of Estimate Std. and Std. Error. Adj-p: p-value of Wald test adjusted for multiple testing.
